# Baseline factors predictive of serious suicidality at follow-up: findings focussing on age and gender from a community-based study

**DOI:** 10.1186/1471-244X-10-41

**Published:** 2010-06-09

**Authors:** A Kate Fairweather-Schmidt, Kaarin J Anstey, Agus Salim, Bryan Rodgers

**Affiliations:** 1 Freemasons Foundation Centre for Men's Health, The University of Adelaide, Adelaide, 5005, South Australia; 2 Centre for Mental Health Research, The Australian National University, Canberra, 0200, Australian Capital Territory; 3 Department of Epidemiology and Public Health, Yong Loo Lin School of Medicine, National University of Singapore, 16 Medical Drive, 117597, Singapore; 4 Australian Demographic & Social Research Institute, The Australian National University, Canberra, 0200, Australian Capital Territory

## Abstract

**Background:**

Although often providing more reliable and informative findings relative to other study designs, longitudinal investigations of prevalence and predictors of suicidal behaviour remain uncommon. This paper compares 12-month prevalence rates for suicidal ideation and suicide attempt at baseline and follow-up; identifies new cases and remissions; and assesses the capacity of baseline data to predict serious suicidality at follow-up, focusing on age and gender differences.

**Methods:**

6,666 participants aged 20-29, 40-49 and 60-69 years were drawn from the first (1999-2001) and second (2003-2006) waves of a general population survey. Analyses involved multivariate logistic regression.

**Results:**

At follow-up, prevalence of suicidal ideation and suicide attempt had decreased (8.2%-6.1%, and 0.8%-0.5%, respectively). However, over one quarter of those reporting serious suicidality at baseline still experienced it four years later. Females aged 20-29 never married or diagnosed with a physical illness at follow-up were at greater risk of serious suicidality (OR = 4.17, 95% CI = 3.11-5.23; OR = 3.18, 95% CI = 2.09-4.26, respectively). Males aged 40-49 not in the labour force had increased odds of serious suicidality (OR = 4.08, 95% CI = 1.6-6.48) compared to their equivalently-aged and employed counterparts. Depressed/anxious females aged 60-69 were nearly 30% more likely to be seriously suicidal.

**Conclusions:**

There are age and gender differentials in the risk factors for suicidality. Life-circumstances contribute substantially to the onset of serious suicidality, in addition to symptoms of depression and anxiety. These findings are particularly pertinent to the development of effective population-based suicide prevention strategies.

## Background

In an effort to reduce prevalence of suicide and suicidal behaviours, many countries have mounted public health campaigns, such as the Australia's National Suicide Prevention Strategy[1]. The Australian Bureau of Statistics (ABS) documents all deaths due to suicide nationwide, and has recently published trends revealing a notable downturn in suicide deaths, most significant among young males[2]. Johnstone *et al.*[3] highlight that although it may be possible to acquire state-administered datasets that allow for disaggregation, the ABS does not administer central database records for non-fatal injuries (including attempted suicides) presenting to Accident and Emergency as, for instance, maintained by The Centers for Disease Control and Prevention in the United States of America. Further, Australian data are event-based, not person-based, which results in difficulties in the calculation of population-based prevalence statistics[3]. These constraints present difficulties for those examining prevalence of non-fatal suicidal behaviour for a corresponding rate attenuation. As a partial consequence of lacking these data, there are no published studies in Australia that have longitudinally mapped rates of suicidal behaviour (as opposed to completed suicides) over time. This contributes to the difficulty in gauging the effectiveness of Australia's National Suicide Prevention Strategy (NSPS; LIFE framework) specifically in terms of non-fatal suicidality. Nonetheless, in a commentary paper reviewing the effect of the NSPS, Robinson *et al.*[4] suggest that the approach may be underperforming due to a lack of specificity.

Moscicki[5] provides a comprehensive review of general risk factors, however Fairweather *et al.*[6] highlight that some variables have a better predictive capacity within certain age or gender groups. This paper extends these works though epidemiologic longitudinal analysis by providing insight into whether these variables, predictive of suicidal behaviour, impact distally[7].

Epidemiological research using community-based surveys avoid bias problematic for investigations involving patient samples, providing more accurate profiles of suicidality in the wider population[8]. General population studies access particular individuals (e.g., suicidal) who may not otherwise be identified, providing valuable information about the community at large and facilitating targeted prevention and intervention programs[9,10]. Despite recently published papers utilising cohort population-based methods[11,12], these remain relatively scarce in suicidological research. Longitudinal designs are able to report incidence rates; measure change within individuals; and, overcome the impact of age differences upon cohort effects by sampling multiple age cohorts[13]. No longitudinal investigation, however, has sought to identify factors measured at baseline that are subsequently associated with the emergence of serious suicidal behaviour (i.e., ideation-plans-attempts) at follow-up specific for age-by-gender groups. The major focus on both life span and gender characteristics is anticipated to yield more targeted information relevant for population-based prevention and intervention programs.

The present study has two objectives. First, to compare annual prevalence rates for suicidal ideation and suicide attempts at baseline and four years later; and, to compare new cases of and remission from serious suicidality (i.e., suicidal ideation, suicide plans, or suicide attempts). Second, to investigate variables measured at baseline (demographics, employment status, mental and physical health, personality, life stresses or social environment factors) that predict serious suicidality four years later for the total sample, and more specifically, separate age-by-gender groups.

## Methods

### Participants and procedure

**Figure 1:**
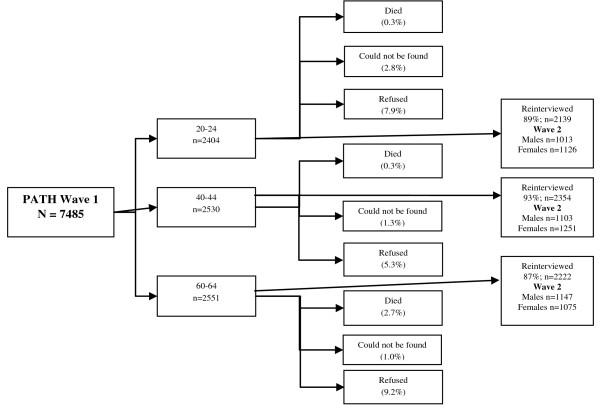
**Flowchart showing participation rates for PATH Wave 1 and Wave 2**.

The sample constitutes participants from both Wave 1 and Wave 2 of the PATH (Personality and Total Health) Through Life Project. For Wave 1 (commenced 1999, completed 2001), participation rate was 58.6% for those aged 20-24 (the 20s group), 64.4% for 40-44 year olds (the 40s group) and 58.3% for 60-64 year olds (the 60s group). Wave 2 (commenced 2003, completed 2006) maintained 89.0% of the 20s group, 93.0% of the 40s group, and 87.1% of the 60s group. At Wave 1 there were 1,009 males and 1,119 females in the 20s group, 1,098 males and 1,246 females in the 40s group, and 1,134 males and 1,060 females in the 60s group. Figure 1 provides a flowchart detailing participation rate for Wave 1 and 2 of the PATH survey. Approval of The PATH Through Life Project protocol (No. M9807) was granted by The Australian National University Human Research Ethics Committee on 22^nd ^September 1998. Survey methodology has been published previously[14].

### Measures

Sociodemographic variables involved current marital status (married/de facto, separated/divorced/widowed, never married), employment status (full-time, part-time, not in labour force), education (total years studying to highest qualification), parent (yes/no). Health and substance use was assessed by the Goldberg Depression and Anxiety Scales[15], the AUDIT scale evaluated alcohol use (abstain, occasional/light, medium, hazardous/harmful[16]), current tobacco smoker (yes/no)[17], and the frequency of marijuana usage was determined (don't use, once or twice per year, once every 1-4 months, once or more per week[18]). Physical health items established whether participant suffered from common chronic diseases[19]. A low prevalence of physical medical conditions necessitated the creation of a single binary variable indicating whether participants had been diagnosed with heart trouble, cancer, arthritis, or diabetes. Relationships and life stressor variables constituted participants' experiences of childhood adversity [20], the number of life events in the last 6 months [21], and two measures of negative interactions; one concerning family, and the other, friends[22]. The personality scales were Eysenck's Psychoticism (EPQ-P) scale and perceived level of mastery[23,24]. The outcome variable ascertained whether respondents had experienced *serious *suicidality. Serious suicidality was indicated by reporting experience of at least one of the following suicidal thoughts or behaviours during the past year: "Have you ever thought about taking your own life"; "Have you made any plans to take your own life"; and "In the last year have you ever attempted to take your own life?"[25].

### Data analysis

#### Descriptive statistics

Comparisons of baseline and follow-up sociodemographic characteristics were undertaken separately for age group and compared within and between genders. Analysis of continuous variables required One-way Analysis of Variance (ANOVA); Pearson's Chi-Square (χ^2^) test with Adjusted Residuals was utilised for categorical variables (SPSS Version 12). McNemar's Test determined significance of follow-up variation in suicidal ideation and suicide attempt prevalence at baseline.

'New suicidality' encompassed participants reporting no serious suicidality at baseline, but serious suicidality at follow-up. 'Remission' comprised serious suicidality at baseline, but not at follow-up. Continued serious suicidality and no serious suicidality included those who report serious suicidality at both or neither data collection points, respectively.

#### Inferential statistics

Participants reporting experience of suicidality during the 12-months prior to baseline were omitted (n = 609). Binary multivariate logistic regression (SPSS Version 12) predicted serious suicidality at follow-up from simultaneously-entered variables associated with suicidality at baseline in those without previous suicidality. The predictor variables comprised age group, gender, marital status, employment status, years of education to highest qualification, frequency of marijuana use, frequency of alcohol use, mastery, childhood adversity, physical medical condition, depression and anxiety, and life events in previous six months. The interaction between age and gender was assessed by entering the term concurrently with all the other predictors.

## Results

### Sociodemographic trends

Significant changes to marital status statistics were apparent at follow-up, as shown in Table 1. More participants were married (46.8% to 53.2%) due to a large proportion of the 20s group marrying after the baseline interview, proportions of separated/divorced/widowed respondents (44.5% to 55.5%) were consistent across age groups, and fewer people remained never married (62.1% to 37.9%). Less people remained in paid employment (51.5% to 48.5%) as a large proportion of the 60s group withdrew from the labour force. The sample continued to spend time in education after the baseline interview (14.2 years to 14.5 years), a statistic mainly driven by the 20s group.

**Table 1:** Unadjusted Comparisons between Wave 1 and Wave 2 participants for age groups within gender sociodemographic characteristics (N = 6,648)

	**Males**					
	
**Age Groups**	**20s**		**40s**		**60s**	
**Wave**	**W1**	**W2**	**W1**	**W2**	**W1**	**W2**
	
**Marital status, %**^**#**^**(AR)**						
Married/de facto	18.3 (-14.9)	49.8	81.9 (0.5)	81.1	88.6 (0.9)	87.4
Sep/div/widowed	0.3 (-5.2)	3.5	9.3 (-1.5)	11.2	9.8 (-0.9)	11.0
Never married	81.4 (16.2)	46.8	8.9 (-1.0)	7.7	1.6 (0.0)	1.6
						
**Employment, %**^**#**^**(AR)**						
Employed	86.2 (-4.1)	91.9	95.7 (2.2)	93.6	50.6 (8.9)	32.2
Not employed	6.5 (2.3)	4.2	1.9 (0.0)	1.9	1.5 (3.5)	0.2
Not in labour force	7.4 (3.3)	4.0	2.4 (-2.7)	4.5	47.9 (-9.5)	67.6
						
**Education mean, (SE) ‡**						
Number of years to highest qualification	14.1 (0.04)	14.7 (0.10)***	14.7 (0.07)	14.9 (0.10)	14.4 (0.08)*	14.6 (0.13)
						
	**Females**					
	
**Age Groups**	**20s**		**40s**		**60s**	
**Wave**	**W1**	**W2**	**W1**	**W2**	**W1**	**W2**
	
**Marital status, %**^**#**^**(AR)**						
Married/de facto	29.1 (-13.2)	56.7	77.4 (1.3)	75.2	69.5 (1.3)	66.8
Sep/div/widowed	1.8 (-4.8)	5.7	15.6 (-1.8)	18.2	27.4 (-1.5)	30.3
Never married	69.1 (8.2)	37.5	7.0 (-0.4)	6.6	3.1 (0.3)	2.9
						
**Employment, %**^**#**^**(AR)**						
Employed	86.1 (-0.3)	86.5	86.1 (-0.3)	86.5	33.3 (7.1)	19.7
Not employed	2.5 (0.7)	2.1	2.5 (0.7)	2.1	0.7 (2.1)	0.1
Not in labour force	10.6 (-0.9)	11.7	11.4 (0.0)	11.4	66.1 (-7.3)	80.2
						
**Education mean, (SE) ‡**						
Number of years to highest qualification	14.4 (0.05)	15.1 (0.12)***	14.3 (0.06)	14.6 (0.12)	13.5 (0.08)	13.8 (0.18)
						

^# ^Percentages are within gender for age group categories

AR = Adjusted residuals; AR > 2 or < - 2 indicates a significant difference between Wave1 and Wave 2 for the respective group; only one AR is reported for each comparison between W1 and W2 which is located in the W1 column.

‡ significance test = One way ANOVA, * p < 0.05, ** p < 0.01, *** p < 0.001.

### Comparison of annual prevalence

Overall, suicidal ideation significantly decreased from baseline to follow-up (8.2% to 6.1%, p < 0.001; Table 2). All age-by-gender categories replicate this downward trend. Similarly, the prevalence of suicide attempt significantly fell (0.8% to 0.5%, p < 0.05), but females aged 40-49 represented the only group to show a notable reduction (1.1% to 0.4%, p < 0.05).

**Table 2:** Annual prevalence rates of suicidal ideation and suicide attempt in the PATH Through Life Project (N = 6,666)

		**Suicidal ideation %**		**Suicide attempts % (n)**	
		
**Gender**	**Age Group**	**Wave 1**	**Wave 2**	**Wave 1**	**Wave 2**
		
**Total**		8.2 (609)	6.1*** (406)	0.8 (60)	0.5 * (34)
**Males**	20s	12.6 (145)	9.3** (94)	1.2 (14)	1.0 (10)
	40s	8.9 (105)	6.3*** (69)	0.7 (8)	0.5 (5)
	60s	3.9 (51)	2.6** (30)	0.2 (2)	0.1 (1)
**Females**	20s	13.4 (165)	9.9** (110)	1.6 (20)	1.0 (11)
	40s	8.8 (117)	6.8** (85)	1.2 (16)	0.4* (5)
	60s	2.1 (26)	1.7 (18)	0	0.2 (2)

Significant difference between Wave 1 and 2, * p < 0.05, ** p < 0.01, *** p < 0.001, McNemar's Test.

### New serious suicidality and remissions

Follow-up data provided the opportunity to record numbers of participants indicating new, and remissions from serious suicidality (see Table 3). At follow-up 3.4% (n = 226) of the sample reported new occurrences of serious suicidality, while 2.7% (n = 179) continued to experience serious suicidality. However, 5.2% of the PATH sample indicated no serious suicidality currently occurred, and the vast majority re-interviewed participants had no serious suicidality at baseline or follow-up (88.7%, n = 5,915). Table 3 shows that, overall, experience of serious suicidality was highest among females aged 20-29, whereas females in their 60s had fewest reports of serious suicidality (no suicidality: 97.0%, n = 1,026).

**Table 3:** New, continued, and remission from serious suicidality at follow-up (N = 6,666)

		**Serious Suicidality**			
	
**Gender**	**Age Group**	**New suicidality % (n)**	**Remission % (n)**	**Continued suicidality % (n)**	**No suicidality % (n)**
	
**Total**		3.4 (226)	5.2 (346)	2.7 (179)	88.7 (5915)
**Males**		3.2 (104)	5.2 (167)	2.7 (88)	88.9 (2882)
	20s	5.3 (53)	7.8 (78)	4.1 (41)	82.9 (837)
	40s	3.1 (34)	5.6 (61)	3.1 (34)	88.2 (969)
	60s	1.5 (17)	2.5 (28)	1.1 (13)	94.9 (1076)
**Females**		3.6 (122)	5.2 (179)	2.7 (91)	88.6 (3033)
	20s	5.5 (60)	8.8 (97)	4.5 (50)	81.3 (912)
	40s	4.0 (49)	5.5 (68)	2.9 (36)	87.7 (1093)
	60s	1.2 (13)	1.3**(14)	0.5**(5)	97.0**(1028)

Significant difference between males and females (total, 20s, 40s, and 60s), * p < 0.05, ** p < 0.01, *** p < 0.001, Chi-square Test.

### Prediction of serious suicidality

After excluding participants who reported suicidality during the 12-months prior to baseline, baseline-measured variables were entered simultaneously into a binary multivariate logistic regression model in which serious suicidality at follow-up comprised the outcome measure. Importantly, there were significant age-related differences in the proportions of participants omitted by this process (25.7% for 20s group, 19.1% for 40s group, and 9.7 for 60s group; χ^2^[2] = 189.9, p < 0.0001).

Results showed a significant main effect for marital status (Wald χ^2^[2] = 9.03, p < 0.05), with participants developing serious suicidality after baseline more likely to be divorced/separated/widowed (OR = 1.70, 95% CI = 1.13, 2.27), or never married (OR = 2.07, 95% CI = 1.50, 2.65). These participants had also greater odds of encountering adversity in their childhood (OR = 1.11, 95% CI = 1.04, 1.17), and experiencing higher levels of depression/anxiety (OR = 1.10, 95% CI = 1.05, 1.14). The sample was split into age-by-gender groups as the interaction was previously found to be significant[6,26].

### Age and gender

Among the males 20s group, females 20s and 60s group depression/anxiety significantly predicted serious suicidality (OR = 1.14, 95% CI = 1.02, 1.26; OR = 1.09, 95% CI = 1.00, 1.18; OR = 1.28, 95% CI = 1.04, 1.52, respectively; Table 4). Other significant predictors appeared more group-specific. Females aged in their 20s had notably higher odds of suicidal behaviour if suffering a physical medical condition (OR = 3.18, 95% CI = 2.09, 4.26) or not married at baseline (OR = 4.17, 95% CI = 3.11, 5.23). When not in the labour force at baseline, the males 40s group had greater odds of subsequent serious suicidality (OR = 4.08, 95% CI = 1.68, 6.48).

**Table 4:** Prediction of serious suicidality at follow-up among participants reporting no suicidality at baseline (N = 6,057)

	Males OR (95% CI)		
	
Variables entered	20s	40s	60s
**Demographics**			
Marital status			
Married/partnered (ref)	1.00	1.00	1.00
Divorced/Separated/Widowed	^	0.62 (0.0, 2.82)	4.29 (0.0, 8.75)
Never married	1.33 (0.23, 2.44)	0.83 (0.0, 3.51)	^
Years studied to highest qualification	0.73 (0.44, 1.02)	1.10 (0.88, 1.32)	0.99
Parent of (a) child(ren)	0.52 (0.0, 2.70)	1.35 (0.0, 3.20)	^
Employment			
Employed	1.00	1.00	1.00
Not employed	1.28 (0.0, 2.67)	^	^
Not in labour force	0.52 (0.0, 2.62)	4.08** (1.68, 6.48)	0.49 (0.0, 1.63)

**Relationships and Life Stressors**			
Number of life events	1.12 (0.88, 1.36)	1.13 (0.73, 1.52)	1.24 (0.0, 2.51)
Childhood adversity	1.08 (0.88, 1.27)	0.90 (0.66, 1.14)	1.08 (0.0, 2.21)
Negative interactions with friends	1.05 (0.83, 1.27)	1.20 (0.88, 1.53)	0.89 (0.0, 1.79)
Negative interactions with family	0.90 (0.69, 1.11)	1.09 (0.82, 1.37)	0.95 (0.0, 1.91)

	**Females OR (95% CI)**		
	
**Variables entered**	**20s**	**40s**	**60s**

**Demographics**			
Marital status			
Married/partnered (ref)	1.00	1.00	1.00
Divorced/Separated/Widowed	^	1.75 (0.0, 3.62)	2.83 (0.50, 5.17)
Never married	4.17*** (3.11, 5.23)	1.05 (0.0, 2.58)	^
Years studied to highest qualification	1.08 (0.83, 1.33)	1.01 (0.0, 2.19)	0.82 (0.40, 1.25)
Parent of (a) child(ren)	1.59 (0.39, 2.80)	0.40 (0.0, 1.57)	^
Employment			
Employed	1.00	1.00	1.00
Not employed	0.63 (0.0, 2.76)	^	^
Not in labour force	2.12 (0.93, 3.31)	0.79 (0.0, 2.12)	0.45 (0.0, 2.10)

**Relationships and Life Stressors**			
Number of life events	1.09 (0.85, 1.33)	0.89 (0.0, 1.91)	0.91 (0.06, 1.75)
Childhood adversity	1.06 (0.89, 1.22)	1.10 (0.0, 2.43)	1.33 (0.98, 1.68)
Negative interactions with friends	1.00 (0.78, 1.23)	0.84 (0.0, 1.77)	0.93 (0.41, 1.45)
Negative interactions with family	0.88 (0.70, 1.06)	0.95 (0.0, 2.04)	1.13 (0.70, 1.57)

	**Males OR (95% CI)**		
	
**Variables entered**	**20s**	**40s**	**60s**

**Health & Substance use**			
Physical medical condition	1.96 (0.34, 3.57)	0.82 (0.0, 2.15)	0.61 (0.0, 1.75)
Depression & Anxiety	1.02 (0.91, 1.12)	1.14* (1.02, 1.26)	1.08 (0.0, 2.26)
Current smoker	0.71 (0.0, 1.59)	0.83 (0.0, 2.20)	1.05 (0.0, 2.72)
Marijuana use			
Don't use (includes previous users; reference group)	1.00	1.00	1.00
Once or twice per year	1.88 (0.92, 2.85)	^	^
Once every one to four months	1.22 (0.0, 2.46)	^	^
At least once per week	1.60 (0.31, 2.90)	^	^
AUDIT^†^			
Abstain	1.00	1.00	1.00
Occasional/light drinking	0.61 (0.0, 1.92)	0.75 (0.0, 2.38)	1.06 (0.0, 2.78)
Medium level drinking	0.76 (0.0, 2.37)	1.42 (0.0, 3.21)	1.19 (0.0, 3.21)
Hazardous/harmful drinking	2.00 (0.25, 3.74)	1.50 (0.0, 4.15)	^

**Personality**			
Psychoticism	1.03 (0.81, 1.24)	1.06 (0.77, 1.34)	1.37 (0.0, 2.90)
Mastery	0.96 (0.84, 1.09)	1.00 (0.85, 1.16)	0.97 (0.0, 2.01)

	**Females OR (95% CI)**		
	
**Variables entered**	**20s**	**40s**	**60s**

**Health & Substance use**			
Physical medical condition	3.18*** (2.09, 4.26)	1.45 (0.0, 3.01)	0.43 (0.0, 2.32)
Depression & Anxiety	1.09* (1.00, 1.18)	1.01 (0.0, 2.26)	1.28* (1.04, 1.52)
Current smoker	0.98 (0.12, 1.84)	1.39 (0.0, 2.92)	0.86 (0.0, 3.55)
Marijuana use			
Don't use (includes previous users; reference group)	1.00	1.00	1.00
Once or twice per year	1.03 (0.06, 2.00)	3.60 (0.0, 7.54)	^
Once every one to four months	0.68 (0.0, 2.22)	^	^
At least once per week	1.72 (0.0, 3.45)	^	^
AUDIT^†^			
Abstain	1.00	1.00	1.00
Occasional/light drinking	1.00 (0.0, 2.17)	0.83 (0.0, 2.20)	1.23 (0.0, 3.38)
Medium level drinking	0.57 (0.0, 2.25)	0.98 (0.0, 2.52)	0.84 (0.0, 3.54)
Hazardous/harmful drinking	^	0.61 (0.0, 2.57)	^

**Personality**			
Psychoticism	1.12 (0.85, 1.39)	0.83 (0.0, 1.74)	1.61 (0.92, 2.30)
Mastery	1.04 (0.91, 1.17)	0.89 (0.0, 1.95)	0.69 (0.26, 1.12)

**OR**: Odds Ratios, 95% Confidence Interval, * p < 0.05, ** p < 0.01, *** p < 0.001

^parameter not available due to small cell size

^†^AUDIT is the Alcohol Use Disorders Identification Test

## Discussion

Although longitudinal methodology confounds developmental age changes with period effects, and comparisons between age groups confound developmental age variation with cohort differences[27], there are many advantages of this approach[28]. These include the capacity to compare baseline and follow-up rates of suicidal ideation, and suicide attempt and provide insight into the influence of distal predictor impact on becoming seriously suicidal.

### Prevalence and trends

Annual prevalence rates of suicidal ideation fell from 8.2% to 6.1%, although the decline among the 60s group was not significant. Further, while overall suicide attempts significantly reduced from 0.8% to 0.5% at follow-up, only the females 40s group reported notably fewer attempts over time. Though it is likely that attrition bias resulted in Wave 2 rates being underestimated, feasible interpretations of the overall decrease in suicidality may encompass the PATH project acting as an intervention, motivating participants to visit their doctor[29], or an overall effect of participants ageing (akin to rates of depression decreasing with age). Other plausible explanations encompass the reduced levels of suicidality being artefactual, as there is the potential for participants to present themselves more positively at re-test[30-32]; and, the National suicide prevention strategies functioning to produce the apparent decline in rates [7].

The analysis of new suicidality showed approximately one third of the male 20s and the female 60s groups reporting serious suicidality were new occurrences. Table 3 clearly illustrates that the youngest cohort has the largest proportion of 'new suicidality', and the largest proportion of 'remissions'. Putatively, for many young adults, active suicidality occurs in response to an acute stressor[33,34]. If the crisis is resolved, or the individual learns to cope with their new reality, suicidal cognitions and behaviours generally dissipate[35]. Nevertheless, some participants experience their suicidality on a continual basis, perhaps co-morbidly with another mental health problem such as depression or anxiety[11]. Rates for suicidality echo trends found for depression/anxiety: decrease with age, and accord with existing literature[26,36] (see Table 2).

### Prediction of serious suicidality at follow-up

The regression model adjusted for the influence of other covariates, tested for interactions between age and gender, and revealed the need for separate age-by-gender models. Analysis conducted on the full sample indicated divorced/separated/widowed participants, never married (and not partnered) at baseline participants, those with more difficult childhoods, and with greater levels of depression/anxiety were all more likely to report serious suicidality four years later. These findings are consistent with existing literature[8,9,11,12], but longitudinal data extend current knowledge. Results suggest that the aforementioned variables remain risk factors in adults throughout the life course, even in the absence of suicidal symptoms. This investigation revealed no main effect for age, most likely a result of the greater prevalence of ideation among young PATH participants at baseline, who were subsequently excluded from the analysis. The significant age-by-gender interaction in the current study affirm recent investigations[6,26] that highlight benefits of considering suicidality by age and gender categories. Some overlap with the total sample was evident, however, analyses of age-by-gender sub-groups revealed several highly specific predictors of serious suicidality. Noteworthy findings will be discussed by the relevant predictor category.

#### Demographics

Previous research concords with the present findings indicating those never married (nor partnered) have increased probability of experiencing serious suicidality[37,38]. However, this analysis further stresses the association between being unpartnered and subsequent serious suicidal behaviour among unpartnered young females. Indeed, this lack of partnership may be felt keenly as many of their similarly-aged counterparts are in relationships, as illustrated by Table 1. It is also possible that the inflated odds of suicidal behaviour in young, never married females are symptomatic of insufficient social support[37,39]. Casey *et al.'s*[40] research more broadly validates the present findings as they found participants from a general population sample with 'people to count on' or were 'shown concern by others' were one-third and two-thirds less likely have suicidal thoughts, respectively.

A particularly noteworthy finding relates to the males 40s group not previously in the labour force nor suicidal at baseline experiencing a four-fold increase in serious suicidality at follow-up. Fairweather *et al. *[6] identified a nine-fold increase in suicide attempts among unemployed, ideating 40-44 year olds, but the present longitudinal methodology shows that non-participation in employment predates suicidality. This investigation emphasises the salience of employment as a protective factor against the development of suicidality in this group. Putatively, being employed is vital to males in their 40s for a number of reasons including providing financial support to their (often young) families, playing an important role in establishing and promoting a sense of male identity and purpose in life[41], and, the work place may afford males with social support and contact[42], shown to be vital in times of stress.

#### Health and substance use

Overall, depression/anxiety robustly predicted serious suicidality at follow-up. In addition to middle-aged males, the female 20s and 60s groups showed a greater likelihood of serious suicidality if initially suffering depression/anxiety. This emphasises the major role of depression/anxiety in subsequent manifestations of suicidal behaviours. While consistent with existing literature[43-45], this analysis highlights the distal relationship between depression/anxiety and suicidality, underscoring the need for prompt diagnosis and treatment of affective syndromes ahead of further stressors/events potentially triggering suicidal behaviour.

The majority of investigations considering physical ill health in relation to suicidality adjust for age and/or gender, utilise samples with greater mean ages[46,47] and commonly focus on completed suicides[48]. Two rare community-based cohort studies (utilising baseline data) indicate that likelihood of suicidal behaviour is significantly elevated among older persons suffering physical illness. De Leo *et al.'s*[46] European-wide study found 20% of those reporting suicidal behaviour when suffering a physical illness or disability indicate that their ill health had a major role in activating their suicidality. Fairweather *et al.*[6] identified that male suicide ideators with physical medical conditions were more likely to attempt suicide than their physically-well counterparts. Uniquely, this paper finds young females reporting no suicidality at baseline, but suffering physical medical conditions (including cancer), experience serious suicidality at threefold the physically well rate at follow-up. The impact of physical illness was larger than symptoms of depression/anxiety. Physical illness functioning as a distal risk may reflect a deterioration in quality of life over time (e.g., as cancer advances), or an increase in pain levels[49,50]. Nevertheless, low cell numbers require this interpretation to be viewed cautiously.

### Strengths and limitations

The design of this investigation has a number of noteworthy strengths including longitudinal and the PATH survey methodology, the large sample, and equivalent proportions of both gender and age cohorts. However, aside from the longitudinal study confounds, limitations include the potential for participants who reported no suicidality in the previous 12 months, to have experienced suicidality prior to this period. It is possible that some individuals were considered non-ideators and consequently included in the baseline sample of non-ideator/plan/attempters. In addition to the survey having restricted age bands, there were three years dividing the data collection points and some categories had small cell size potentially impacting the capacity to detect effects. The information provided was also retrospective and self-reported.

## Conclusions

Although follow-up prevalence rates of suicidal ideation, suicide attempt and other statistics concerning serious suicidality provide valuable information, the main focus of the paper was to identify factors predictive of serious suicidality at follow-up among those who initially reported no suicidality. This investigation demonstrates the presence of age and gender differences in factors distally predictive of serious suicidality. Consideration of these basic demographic characteristics may help to focus suicidal symptom identification in clinical settings, and contributes to the level of specificity that prevention and intervention programs are currently argued to be lacking. Future research opportunities remain to be explored which take into account change in the proximal predictors of suicidality and the presence of suicidality.

## Competing interests

The authors declare that they have no competing interests.

## Authors' contributions

All authors have read and approved the final manuscript. AKF-S conceived the study, performed the majority of the statistical analysis and drafted the manuscript. KJA was involved in critically revising the manuscript for important intellectual content and data acquisition. AS performed an essential component of the data analysis, and contributed to the method section. BR critically reviewed the manuscript and was also involved in data acquisition.

## Pre-publication history

The pre-publication history for this paper can be accessed here:

http://www.biomedcentral.com/1471-244X/10/41/prepub
